# Molecular Mechanisms Explaining Neuroanatomical Subtypes in Major Depressive Disorder: Insights From Cortical Morphometric Inverse Divergence

**DOI:** 10.1002/hbm.70383

**Published:** 2025-10-31

**Authors:** Yao Ge, Lijuan Chen, Yan Bai, Wei Wei, Yu Shen, Kaixin Li, Mengzhu Wang, Meiyun Wang

**Affiliations:** ^1^ Department of Radiology Zhengzhou University People's Hospital & Henan Provincial People's Hospital Zhengzhou China; ^2^ MR Research Collaboration, Siemens Healthineers Ltd. Beijing China; ^3^ Biomedical Research Institute, Henan Academy of Sciences Zhengzhou China

**Keywords:** gene expression, major depressive disorder, morphometric inverse divergence network, neuroanatomical heterogeneity, neurotransmitter

## Abstract

Major depressive disorder (MDD) exhibits substantial neurobiological heterogeneity that complicates treatment selection and mechanistic understanding. While conventional group‐level analyses identify diverse structural alterations, they obscure clinically relevant individual differences. We employed heterogeneity through discriminant analysis (HYDRA) clustering to decompose morphometric inverse divergence (MIND) network patterns into distinct neuroanatomical subtypes and examined their molecular underpinnings. We analyzed MIND network data from 240 Japanese individuals with MDD and 367 healthy controls using unsupervised clustering. Subtype‐specific alterations were mapped onto neurotransmitter receptor density distributions, and transcriptomic data from the Allen Human Brain Atlas were integrated using partial least squares regression. Two neuroanatomically distinct subtypes emerged. Subtype 1 (*n* = 78) exhibited widespread increases in MIND strength across all Yeo networks, with predominant serotonergic, dopaminergic, and GABAergic associations. Gene expression analysis revealed SST and CUX2 correlations, with enrichment for metal ion homeostasis and circadian rhythm pathways. Subtype 2 (*n* = 162) showed reduced MIND strength in dorsal attention, somatomotor, frontoparietal, limbic, and default networks, with glutamatergic, cannabinoid, and dopaminergic dysfunction. This subtype demonstrated negative CRH correlations and enrichment for glutamatergic signaling and calcium/cAMP‐mediated processes. Our findings demonstrate systematic decomposition of MDD heterogeneity into distinct neuroanatomical subtypes with unique molecular signatures. The identification of subtype‐specific neurotransmitter profiles and transcriptomic architectures provides mechanistic insights into MDD heterogeneity, offering potential for biomarker‐guided treatment selection and personalized therapeutic approaches.

## Graphical abstract

MIND‐based clustering identified two MDD subtypes with distinct molecular signatures.

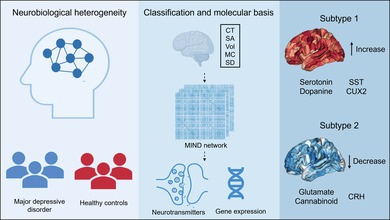

## 1 Introduction

Major depressive disorder (MDD) is a widespread psychiatric condition marked by a consistently low mood and diminished interest or enjoyment in everyday activities, exerting a profound impact on the global mental health burden (Simon et al. 2024). The heterogeneity of MDD poses significant challenges to diagnosis and treatment, as traditional symptom‐based classification systems fall short in capturing the underlying neurobiological diversity (Buch and Liston 2021). Consequently, therapeutic approaches often lack precision, leading to varied efficacy and leaving 30%–40% of patients inadequately treated despite antidepressant or psychotherapeutic interventions (Veal et al. 2024). This phenotypic diversity has profound implications for diagnosis, prognosis, and therapeutic intervention, highlighting the critical need to identify biologically meaningful subtypes that can inform precision medicine approaches in psychiatry.

Previous neuroimaging studies have identified structural and functional brain abnormalities in patients with MDD (Brandl et al. 2022; Goodkind et al. 2015). These macroscale structural and functional changes demonstrate regional specificity, with the evidence supporting alterations in the dorsolateral prefrontal cortex and orbitofrontal cortex regions implicated in depressive symptoms and cognitive function (Gray et al. 2020). However, substantial variability exists in reported findings, with conflicting evidence regarding directionality and spatial distribution of the changes across independent cohorts (Beijers et al. 2019; Winter et al. 2022). For instance, studies have reported that individuals with MDD often exhibited decreased volumes in areas such as the prefrontal cortex (Goodkind et al. 2015). However, other studies have not observed the same reduction (Gray et al. 2020). Abnormalities in functional connectivity, such as the default network, have been repeatedly reported in individuals with MDD, but the specific brain regions where connectivity has been enhanced or attenuated have been inconsistent across studies (Winter et al. 2022). Such discrepancies likely reflect underlying neurobiological heterogeneity, suggesting that conventional case–control analyses may obscure meaningful subtype‐specific patterns embedded within MDD populations (Beijers et al. 2019).

Objective neuroanatomical profiling has revealed distinct subtypes across MDD, with stable neurobiological clustering patterns linked to differential clinical presentations (Beijers et al. 2019). Recent studies have identified discrete MDD subtypes based on contrasting cortical morphometry alterations, highlighting neurobiological heterogeneity within MDD (Lalousis et al. 2022; Li et al. 2024). Nevertheless, existing clustering studies predominantly rely on a single morphometric measure, such as cortical thickness or regional volume, potentially limiting classification sensitivity and biological interpretability. Integrating multiple morphometric parameters through advanced network‐based approaches could substantially enhance subtype identification accuracy. morphometric inverse divergence (MIND) method quantifies cortical structural similarity by computing symmetrized Kullback–Leibler (KL) divergence between regional multivariate distributions of five morphometric features, generating bounded similarity indices with enhanced sensitivity for detecting architectural patterns (Sebenius et al. 2023). Despite its robustness, MIND has yet to be applied to explore the heterogeneity of MDD. Additionally, heterogeneity through discriminative analysis (HYDRA) employs semi‐supervised machine learning to perform binary classification and subtype identification, distinguishing pathological samples from healthy controls (HCs) (Varol et al. 2017). Combining MIND with HYDRA presents a powerful approach for uncovering MDD subtypes and advancing our understanding of its neuroanatomical heterogeneity.

Genetic components serve as significant contributors to MDD etiology (Kendall et al. 2021). Brain‐wide transcriptomic atlases enable systematic investigation of spatial gene expression relationships with structural neuroimaging phenotypes, facilitating mechanistic interpretation of observed neuroanatomical alterations (Hawrylycz et al. 2012). Recently, Li et al. (2024) linked the cortical morphological network of MDD with AHBA gene expression data, revealing a link between macrostructural changes in MDD and synapse‐related terms. Meanwhile, MDD has been strongly associated with several neurochemical pathways, encompassing gamma‐aminobutyric acid (GABA) and serotonin (Javelle et al. 2025; Simmonite et al. 2023), thereby helping to shed light on the molecular and anatomical relationships that underpin MDD heterogeneity.

The present study sought to identify distinct neuroanatomical subtypes within MDD and elucidate the molecular mechanisms underlying their differential morphometric patterns. We hypothesized that MDD patients exhibit heterogeneous cortical alterations that can be clustered into neurobiologically meaningful subtypes, each characterized by specific molecular signatures. Our approach employed HYDRA clustering on MIND‐derived morphometric profiles to identify distinct MDD subtypes based on their patterns of cortical structural deviation. We then systematically characterized the molecular signatures distinguishing these subtypes through neurotransmitter receptor mapping and transcriptomic analysis using Allen Human Brain Atlas (AHBA) data. Through partial least squares (PLS) and functional enrichment analysis, we sought to establish the biological pathways that differentiate the identified subtypes, thereby revealing the molecular mechanisms driving subtype‐specific neuroanatomical alterations in depression.

## 2 Methods and Materials

### 2.1 Participants

We utilized the publicly available magnetic resonance imaging (MRI) dataset derived from the OPEN Strategic Research Program for Brain Sciences (SRPBS) neuroimaging collection (Tanaka et al. 2021), with participant demographics summarized in Table 1. The dataset included 372 HCs and 255 individuals with MDD, recruited from three sites. All the patients with MDD were diagnosed based on the Diagnostic and Statistical Manual of Mental Disorders, Fourth Edition (DSM‐IV) criteria. The Mini‐International Neuropsychiatric Interview (MINI) was used to screen HCs. Further details for each site can be accessed in Table S1. All participants underwent three‐dimensional T1‐weighted (T1w) imaging. The severity of symptoms was evaluated with the Beck Depression Inventory‐II (BDI‐II). Imaging parameters for T1w are outlined in Table S2. Following a detailed image quality review of 20 participants (15 MDD patients and 5 HCs), it was determined that the T1w images for these individuals were of poor quality, leading to their exclusion from subsequent analyses. The remaining dataset, comprising 607 participants (367 HCs and 240 MDD), was used for further analysis.

**TABLE 1:** Demographic and clinical characteristics between MDD subtypes and HC.

Variable	HC (*n* = 367)	MDD (*n* = 240)	*p*	MDD subtype 1 (*n* = 78)	MDD subtype 2 (*n* = 162)	*p*
Sex, female (%)	197 (53.68)	121 (50.41)	0.482^a^	44 (56.41)	77 (47.53)	0.249^a^
Age (years)	43.45 ± 14.56	42.37 ± 12.13	0.324^b^	42.03 ± 9.75	42.53 ± 13.14	0.736^b^
Handedness, right (%)	340 (92.64)	220 (91.67)	0.776^a^	70 (89.74)	150 (92.59)	0.618^a^
TIV	1593.12 ± 115.99	1526.01 ± 155.04	0.124^b^	1492.56 ± 125.45	1530.06 ± 111.44	0.754^b^
BDI‐II		26.58 ± 9.91		25.81 ± 10.10	27.22 ± 9.76	0.404^b^

Abbreviations: BDI‐II, Beck Depression Inventory‐II; HC, healthy control; MDD, major depressive disorder; TIV, total intracranial volume.

^a^ Chi‐square test.

^b^ Two sample *t*‐test.

### 2.2 Imaging Preprocessing

T1w images underwent preprocessing using surface‐based analysis through FreeSurfer v6.0 (Fischl 2012). For all participants, the total intracranial volume (TIV) calculations were performed. In addition, cortical surface reconstruction was performed, which included skull stripping, segmentation of brain tissues, separation of hemispheres, subcortical structure segmentation, and the creation of gray–white matter interfaces and cortical surfaces (Hedges et al. 2022).

### 2.3 Construction of MIND

**FIGURE 1:**
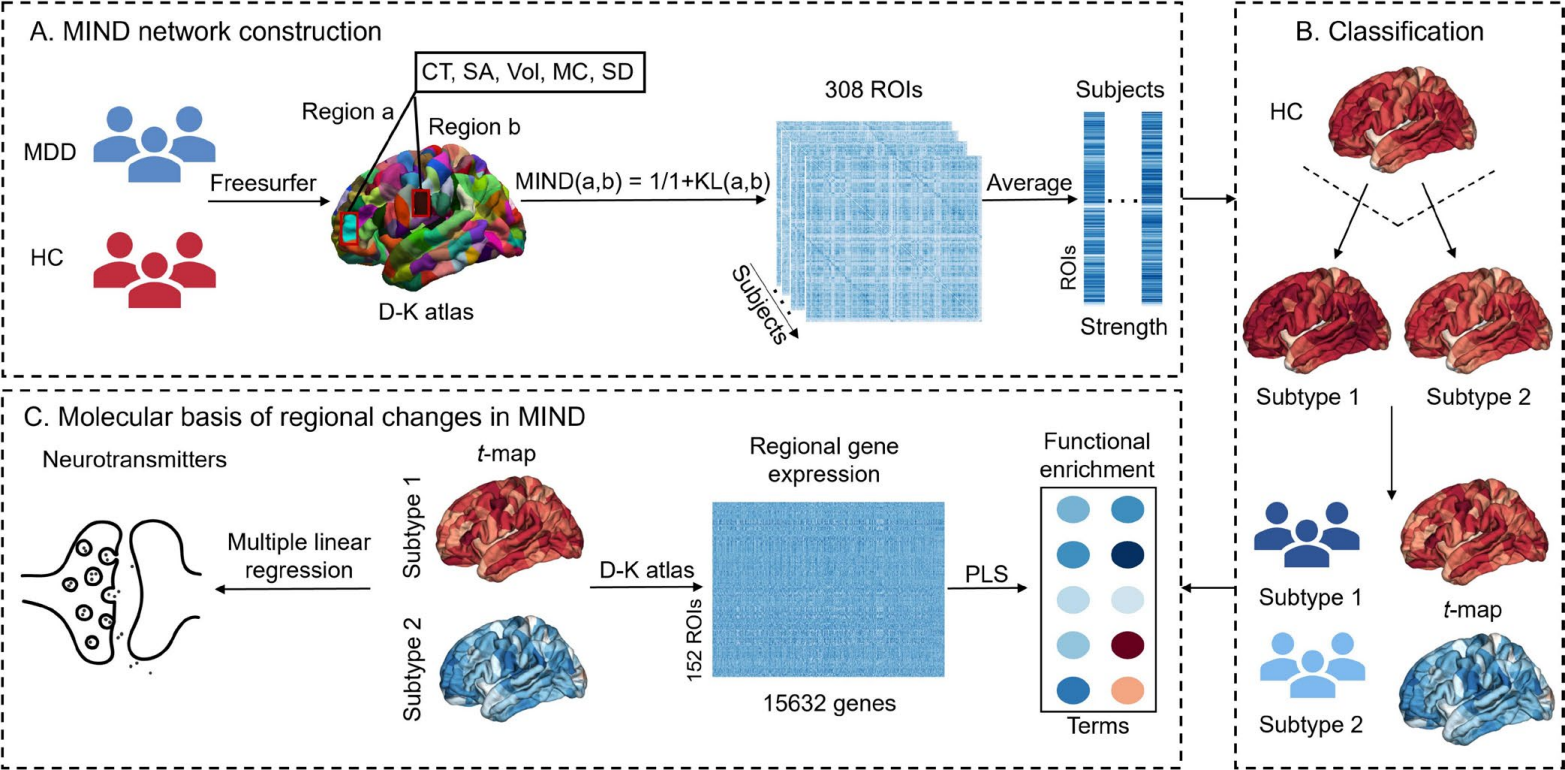
Study overview. (A) MIND network construction. The 308 × 308 matrix for MIND was calculated across multiple macrostructural features (cortical thickness, surface area, gray matter volume, mean curvature, and sulcal depth). The MIND strength was determined by averaging connections across 308 cortical areas without applying a threshold. (B) Classification. The HYDRA technique was applied for detecting distinct MDD subtypes by analyzing variations in MIND strength. (C) Molecular basis of regional changes in MIND. Utilizing multiple linear regression models to identify neuroimaging‐neurotransmitter associations within two MDD subtypes. PLS regression analysis of the gene expression data from the Allen Human Brain Atlas revealed subtype‐associated molecular signatures.

Cortical surface parcellation yielded 308 spatially continuous regions, employing 68 cortical zones from the Desikan–Killiany (D–K) atlas, referred to as the DK‐308 atlas (Romero‐Garcia et al. 2012; Seidlitz et al. 2018). This parcellation method aimed for nearly equal region sizes, utilizing a backtracking algorithm to minimize variability in parcel sizes. The standard surface space was then transformed into each participant's individual space. Each vertex on the reconstructed cortical surface was described by several structural features, such as cortical thickness, surface area, gray matter volume, mean curvature, and sulcal depth (Sebenius et al. 2023). These features were standardized (*Z* score) across all vertices to ensure consistency. For each pair of regions defined by the 308‐parcellation, multivariate distributions of these structural features were computed. To assess the dissimilarity between these distributions, the KL divergence was applied (Sebenius et al. 2023). Subsequently, the 308 × 308 MIND matrix was created for each participant (Figure 1).

For example, a symmetric measure of the KL divergence between regions *a* and *b* was given by the following formula:

$$\hat{D}\left(P_{a},P_{b}\right)=\max\left({\hat{D}}_{\mathrm{KL}}\left(P_{a}∥P_{b}\right),0\right)+\max\left({\hat{D}}_{\mathrm{KL}}\left(P_{b}∥P_{a}\right),0\right)$$

And MIND between region *a* and region *b* was then estimated by:

$$\text{MIND}\left(a,b\right)=\frac{1}{1+\hat{D}\left(P_{a},P_{b}\right)}$$

Additionally, regional MIND strength was computed by averaging connections across 308 cortical areas without applying a threshold (Yao et al. 2024).

### 2.4 Data Harmonization

To obtain reliable mapping of MIND strength differences between MDD patients and HCs, we applied Combat (https://github.com/Jfortin1/ComBatHarmonization) for harmonizing individual MIND strengths across different sites (Fortin et al. 2018, 2017). In this model, we also incorporated the diagnostic label (MDD or HC) as a biological variable of interest, ensuring that the group‐level MIND differences were maintained following harmonization.

### 2.5 MDD Subtyping With HYDRA

HYDRA distinguishes subtypes within the MDD cohort by contrasting MDD patients with HCs (Varol et al. 2017). In contrast to traditional supervised learning approaches like support vector machines and random forests, which are unable to identify subtypes within MDD, HYDRA simultaneously performs classification and clustering. The classification process was performed by separating HCs from MDD subjects through linear maximum‐margin classifiers, generating a polytope structure. For categorization, MDD patients were clustered according to their correspondence with various polyhedron facets, termed hyperplanes. HYDRA processing was executed with the specific parameters: 50 iterations alternating between hyperplane estimation and cluster estimation, 20 consensus steps for clustering, a regularization parameter of 0.25, and 10‐fold cross‐validation (Yao et al. 2023). Additionally, the clustering results' similarity was assessed using the Adjusted Rand Index (ARI), which measured clustering consistency across 10‐fold cross‐validation procedures (Chand et al. 2020). ARI methodology corrects for random variability, delivering stringent overlap quantification.

### 2.6 Reproducibility Validation Analyses

Reproducibility assessment of neurobiological subtypes was rigorously evaluated through three distinct validation methodologies: (i) split‐sample methods evaluated consistency of subtype numbers when data were randomly divided into independent 80%–20% train–test cohorts across 100 iterations (Lange et al. 2004); (ii) split‐half validation assessed reproducibility of regional MIND strength patterns by randomly dividing the dataset into equal halves; and (iii) leave‐one‐site‐out validation examined the stability of classification solutions by systematically excluding individual acquisition centers (Arlot and Celisse 2010).

### 2.7 Case–Control Comparison of MIND Within MDD Subtypes

We fitted the linear regression models to compare the regional MIND strength between the MDD subtypes and HCs' differences, adjusting for age, sex, TIV, and handedness:

$$\begin{array}{c} {\text{MIND}}_{i}=\beta_{0}+\beta_{1}\times \mathrm{Age}+\beta_{2}\times \mathrm{Sex}+\beta_{3}\times \mathrm{TIV}\\ \;+\beta_{4}\times \text{Handedness}+\beta_{5}\times \text{Group}+\varepsilon \end{array}$$

For each region *i*, *MIND*_*i*_ was the MIND strength, age, sex, TIV, and handedness were terms used to adjust for these factors, respectively. Group is the group factor (i.e., MDD subtype 1 and MDD subtype 2). Next, two‐sided *t*‐tests were performed. Furthermore, to provide context for the regional MIND changes, we further categorized 308 regions according to two recognized cortical area categorization systems. The Yeo 7 functional atlas was used to group regions based on resting‐state networks (Yeo et al. 2011), while the von Economo atlas classified regions according to cytoarchitectonic characteristics (von Economo and Koskinas 1925; Whitaker et al. 2016). For each classification, the mean MIND strength was computed across every region within each Yeo network or von Economo classes. Linear regression models were then applied to evaluate the variations in MIND strength between MDD subtypes and control groups, adjusting for the same covariates. Additionally, we investigated the associations between abnormal regional MIND strength and BDI‐II scores among individuals with MDD subtypes, employing partial correlation while controlling for the same covariates. All analyses underwent Bonferroni correction adjustment, establishing significance criteria at *p* < 0.017, which was implemented to adjust for multiple comparisons.

### 2.8 Neurotransmitters Related to MIND

The region‐wise *t* value maps from the MIND changes induced by MDD subtypes were utilized as the input to analyze spatial correlation against the spatial expression maps of receptors and transporters across the brain available in the JuSpace toolbox (https://github.com/juryxy/JuSpace) (Dukart et al. 2021). These maps, obtained through PET and SPECT imaging, cover different neurotransmitter systems and receptors, including the serotonin systems (5HT1a, 5HT1b, 5HT2a, 5HT4, 5HT6, and serotonin transporter [SERT]) (Beliveau et al. 2017; Fazio et al. 2016; Savli et al. 2012), the dopamine systems (D1, D2, DAT, and FDOPA) (Alakurtti et al. 2015; Dukart et al. 2018; Kaller et al. 2017), the GABAergic receptor (Dukart et al. 2018), the N‐methyl‐D‐aspartate (NMDA) receptor (Galovic et al. 2023, 2021), the metabotropic glutamate receptor 5 (mGluR5) (Hansen et al. 2022), the cannabinoid receptor (CB1) (Laurikainen et al. 2019), the μ‐opioid receptor (MU) (Kantonen et al. 2020), the noradrenaline transporter (NAT) (Hesse et al. 2017), the synaptic vesicle glycoprotein 2A (SV2A) transporter (M.‐K. Chen et al. 2018), and the vesicular acetylcholine transporter (VAChT) (Hansen et al. 2022). Standardized beta coefficients were calculated between the *t* map and neurotransmitter maps using multiple linear regression across 308 cerebral cortical regions from the DK‐308 atlas, while accounting for spatial autocorrelation and partial volume effects using the gray matter probability map (Dukart et al. 2021). Exact *p* values were determined through spatial permutation‐based null maps with 10,000 permutations. All analyses were adjusted using a false discovery rate (FDR) correction, with a significance threshold set at *p* < 0.05.

### 2.9 Gene Expression Data Acquisition and Processing

Transcriptomic profiles acquired from six postmortem cerebral samples, encompassing 3702 unique spatial locations, were retrieved from the AHBA dataset (http://human.brain‐map.org) (Hawrylycz et al. 2012). This collection included 58,692 probes corresponding to 20,737 genes, providing comprehensive spatial annotations linking transcriptional information to distinct brain regions. To preprocess the AHBA data, we used the Abagen toolbox (https://github.com/rmarkello/abagen) in accordance with established protocols (Markello et al. 2021). The preprocessing steps included: (i) mapping microarray probes to gene identifiers, (ii) filtering out low‐intensity probes with expression values beneath background noise in greater than 50% of sample instances, (iii) opting for probes exhibiting the greatest regional consistency for genes examined by multiple probes, (iv) categorizing samples into brain regions via 2 mm Euclidean distance proximity to region margins, and (v) standardizing gene expression among tissue samples via scaled sigmoid transformation method. Given the limited right hemisphere data within the AHBA data, we centered our analysis on the left hemisphere (Arnatkeviciute et al. 2019), resulting in a transcriptomic matrix comprising 152 regions and 15,632 gene expression records.

### 2.10 Examining the Relationship Between Transcriptomic Profiles and Regional MIND Differences

We used PLS regression to investigate the spatial associations between the expression levels of all 15,632 genes as predictor variables and regional changes in MIND (*t* value maps from 152 cortical regions) as response variables (Abdi and Williams 2013). The first PLS component (PLS1) or the second component (PLS2) was determined as a linear combination of gene expression, demonstrating the most robust association with *t* value maps. Then, we chose PLS1 as the optimal component due to its highest variance explanation. To evaluate whether the covariance between transcriptomic scores and *t* value maps within the PLS1 component transcended random probability, we implemented a permutation test involving 10,000 iterations. Additionally, we applied bootstrapping to assess the variability of each gene within the PLS1 component (Li et al. 2021; Yao et al. 2023). *Z* values were computed by dividing each region's expression weight by its bootstrap standard error, and genes were ranked according to their contribution to the PLS1 component (Yao et al. 2023). The statistically significant genes were organized into two discrete groups, termed PLS1+ genes (*Z* > 5) and PLS1− genes (*Z* < −5), according to their PLS1 weight measurements (Yao et al. 2023).

To further understand the underlying mechanisms, we examined the connection between regional MIND strength alterations and genes associated with MDD via implementing spatial correlation assessment involving *t* value maps and MDD‐related genes at the in situ hybridization (ISH) level. MDD‐related genes were identified from the AHBA dataset (Hawrylycz et al. 2012), which compiled all available data. The identification of these genes was derived from established literature and encompassed 24 MDD‐related genes (Zeng et al. 2012). These genes were recognized for their involvement in physiological pathways associated with MDD. To delve deeper into their role within PLS analysis, we initially pinpointed genes shared by the MDD‐related gene lists and the 15,632 background genes. Subsequently, we assessed the relationships linking the transcriptomic profiles of these overlapping genes to regional variations in MIND strength within the left hemisphere. A significance threshold of *p* < 0.05 was applied to determine statistical relevance, with FDR correction applied for multiple comparisons.

### 2.11 Functional Enrichment Analysis

To obtain a comprehensive understanding of PLS1 weighted gene functional properties, we utilized the Metascape platform for conducting multiple enrichment analysis investigations (Zhou et al. 2019). These analyses included the examination of biological processes through Gene Ontology (GO) as well as the determination of enriched pathways from the Kyoto Encyclopedia of Genes and Genomes (KEGG). PLS1+ (Z > 5) or PLS1− (Z < −5) values were submitted to the Metascape tool, subsequently evaluating the derived enriched pathways for statistical significance, using a 0.05 threshold with FDR correction (Li et al. 2021).

### 2.12 Null Models

To address the confounding effects of spatial autocorrelation, we utilized spatial autocorrelation‐preserving permutation tests, known as “spin” tests (Váša et al. 2018). This method involved executing 10,000 random spatial rotations of the cortical regions to establish a null distribution. The *p*_spin_ values were then calculated by comparing the observed values to the null distribution, using the < 5th or > 95th percentiles as thresholds. The significance threshold for these spatial permutation tests was at *p*_spin_ < 0.05.

## 3 Results

### 3.1 Demographic Characteristics and Two MDD Subtypes

The demographic and clinical characteristics revealed no significant differences in age, sex, or handedness between HCs and MDD patients. Similarly, there were no notable differences in TIV (Table 1). To assess the consistency of clustering, we varied the number of clusters from 2 to 8 and used the ARI. The highest ARI value was observed at *K* = 2 (ARI = 0.75), indicating that the MDD patients were most effectively categorized into two subtypes based on the MIND strength. At cluster resolutions between *K* = 3 and *K* = 8, ARI values were lower compared to those at *K* = 2. As a result, 78 MDD patients were grouped into subtype 1, and 162 into subtype 2. The results revealed no significant differences in age, sex, handedness, TIV, or BDI‐II scores between subtype 1 and subtype 2 (Table 1).

### 3.2 Multiscale MIND Alterations Delineate Neuroanatomical Subtypes of MDD

**FIGURE 2:**
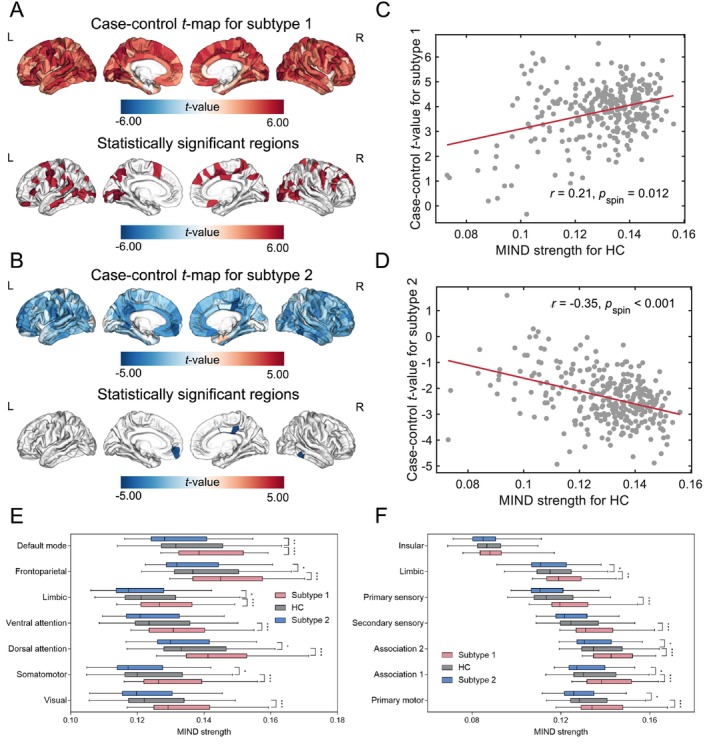
MDD subtypes‐related regional changes in MIND. (A) Case–control comparison of regional MIND strength between MDD subtype 1 patients and HC. Subtype 1 patients showed regional increased MIND. (B) Comparison of regional MIND strength between patients with MDD subtype 2 and HC. In patients with subtype 2, MIND was significantly reduced only in the left medial orbitofrontal, right inferior temporal, and posterior cingulate cortex. (C) A scatterplot of mean control MIND vs. case–control *t* values for subtype 1 indicated a positive spatial correlation with regional MIND strength in HC (*r* = 0.21, *p*_spin_ = 0.012). (D) A scatterplot for subtype 2 presented a negative spatial correlation between case–control *t* values and regional MIND strength in HC (*r* = −0.35, *p*_spin_ < 0.001). (E) Analysis of MIND differences in Yeo functional networks between MDD subtype patients and HC showed that subtype 1 patients had elevated MIND strength across all networks (Bonferroni‐corrected *p* < 0.017), while subtype 2 patients had diminished MIND strength, primarily affecting somatomotor, dorsal attention, limbic, frontoparietal, and default mode networks (Bonferroni‐corrected *p* < 0.017). (F) Examination of MIND variations in von Economo networks indicated that subtype 1 patients had notably increased MIND strength in primary motor, association 1 and 2, secondary sensory, primary sensory, and limbic networks (Bonferroni‐corrected *p* < 0.017). In contrast, subtype 2 patients showed reduced MIND strength in the primary motor, association networks 1 and 2, and limbic networks (Bonferroni‐corrected *p* < 0.017) (*p < 0.017, **p < 0.003, ***p < 0.0003).

The spatial distribution of MIND across brain regions showed similar patterns between the MDD subtypes and HCs (Figure S1). All groups displayed higher values predominantly in the frontal and temporoparietal cortices, while the insular cortex exhibited lower values. We assessed the differences in MIND patterns between each MDD subtype and the HC group, measured by region‐wise MIND strength. For subtype 1, increased MIND strength compared to HCs was detected in both the caudal and rostral regions of the middle frontal gyrus, superior frontal, pars triangularis, precentral gyrus, inferior, middle, and superior temporal, superior and inferior parietal, postcentral, precuneus, supramarginal, lingual, and lateral occipital gyri bilaterally, and pericalcarine cortex (Figure 2A). Conversely, subtype 2 exhibited statistically significant reductions in MIND strength compared to HCs, specifically noted in the left medial orbitofrontal, right inferior temporal gyrus, and right posterior cingulate cortex (Figure 2B). Additionally, we found an increase in MIND strength in subtype 1 compared to subtype 2 across the cortex (Figure S2).

To further analyze the network‐level MIND differences between cases and controls, we categorized cortical areas into two predefined atlases. For the Yeo functional network, patients with MDD subtype 1 exhibited a significant increase in MIND across all seven networks: visual, somatomotor, dorsal attention, ventral attention, limbic, frontoparietal, and default mode networks. However, all patients with MDD subtype 2 exhibited significantly reduced MIND strength, primarily affecting the somatomotor, dorsal attention, limbic, frontoparietal, and default mode networks (Figure 2E). Within the von Economo atlas, patients with MDD subtype 1 displayed an increase in MIND strength in the primary motor, associations 1 and 2, secondary sensory, primary sensory, and limbic networks. However, patients with MDD subtype 2 exhibited reduced MIND strength in the primary motor and association networks 1 and 2, as well as the limbic networks (Figure 2F).

We further analyzed the association of MIND strength within the HC group relative to case–control *t*‐maps for the two subtypes. Subtype 1 displayed a positive spatial correlation (*r* = 0.21, *p*_spin_ = 0.012), while subtype 2 displayed a negative spatial correlation (*r* = −0.35, *p*_spin_ < 0.001) (Figure 2C,D). Furthermore, we evaluated the association between case–control variations in MIND and BDI‐II scores among patients with different MDD subtypes using partial correlation analysis. Nonetheless, no significant associations were detected following Bonferroni correction.

### 3.3 Reproducibility Analysis of MDD Subtypes

The validation of identified MDD subtypes was demonstrated through comprehensive reproducibility testing protocols. Split‐sample validation confirmed exceptional stability of the two‐subtype classification scheme across independent cohort divisions (Figure S3). Regional MIND strength patterns exhibited consistent reproducibility between sample halves during split‐half validation procedures at the optimal two‐cluster solution (Figure S4). Cross‐validation analyses were subsequently implemented using the leave‐one‐site‐out methodology to assess subtype stability across different acquisition centers. When cluster parameters were configured for dual subtype identification, predicted classification labels generated through leave‐one‐site‐out procedures were systematically compared against original assignments derived from complete multisite integration. The concordance rate for patients maintaining identical subtype assignments reached 86.17% (Figure S5), demonstrating substantial reproducibility of the neuroanatomical clustering framework. These validation results confirm the reliability of MIND‐based subtype identification and support the neurobiological validity of the discovered MDD phenotypic heterogeneity patterns.

### 3.4 Associations Between MIND Alterations and Neurotransmitter Distribution

**FIGURE 3:**
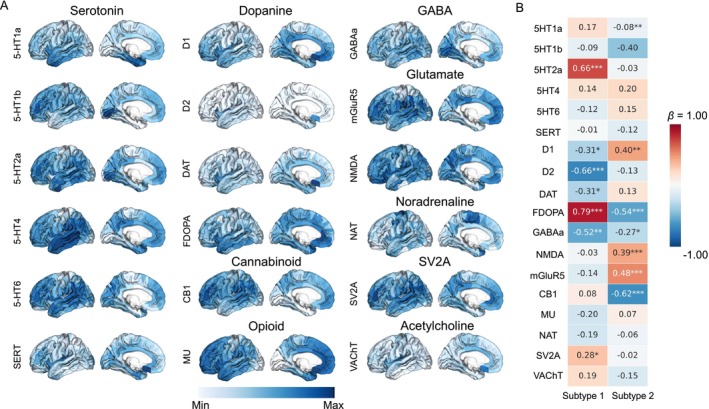
The association between neurotransmitter distribution and changes in MIND. (A) Neurotransmitter systems were mapped with positron emission tomography, resulting in a map of 308 cortical regions. (B) The heatmap displayed the spatial correlation analysis between neurotransmitters and *t*‐maps of patients with MDD subtypes 1 and 2 alongside controls.

Spatial analysis revealed the links between MIND alterations associated with MDD subtypes and neurotransmission systems (Figure 3A,B). Specifically, several neurotransmitters were linked to the case–control *t*‐map of MIND, as indicated by significant associations in MDD subtype 1 after FDR correction: 5‐HT2a (*β* = 0.66, *p* < 0.001), D1 (*β* = −0.31, *p* = 0.017), D2 (*β* = −0.66, *p* < 0.001), DAT (*β* = −0.31, *p* = 0.017), FDOPA (*β* = 0.79, *p* < 0.001), GABAa (*β* = −0.52, *p* = 0.001), and SV2A (*β* = 0.28, *p* = 0.033). Moreover, the case–control *t*‐map for MDD subtype 2 showed significant associations with several neurotransmitters: 5‐HT1a (*β* = −0.08, *p* = 0.005), D1 (*β* = 0.40, *p* = 0.001), FDOPA (*β* = −0.54, *p* < 0.001), GABAa (*β* = −0.27, *p* = 0.036), NMDA (*β* = 0.39, *p* < 0.001), mGluR5 (*β* = 0.48, *p* < 0.001), and CB1 (*β* = −0.62, *p* < 0.001).

### 3.5 Transcriptional Profiles Linked to Regional MIND Differences

**FIGURE 4:**
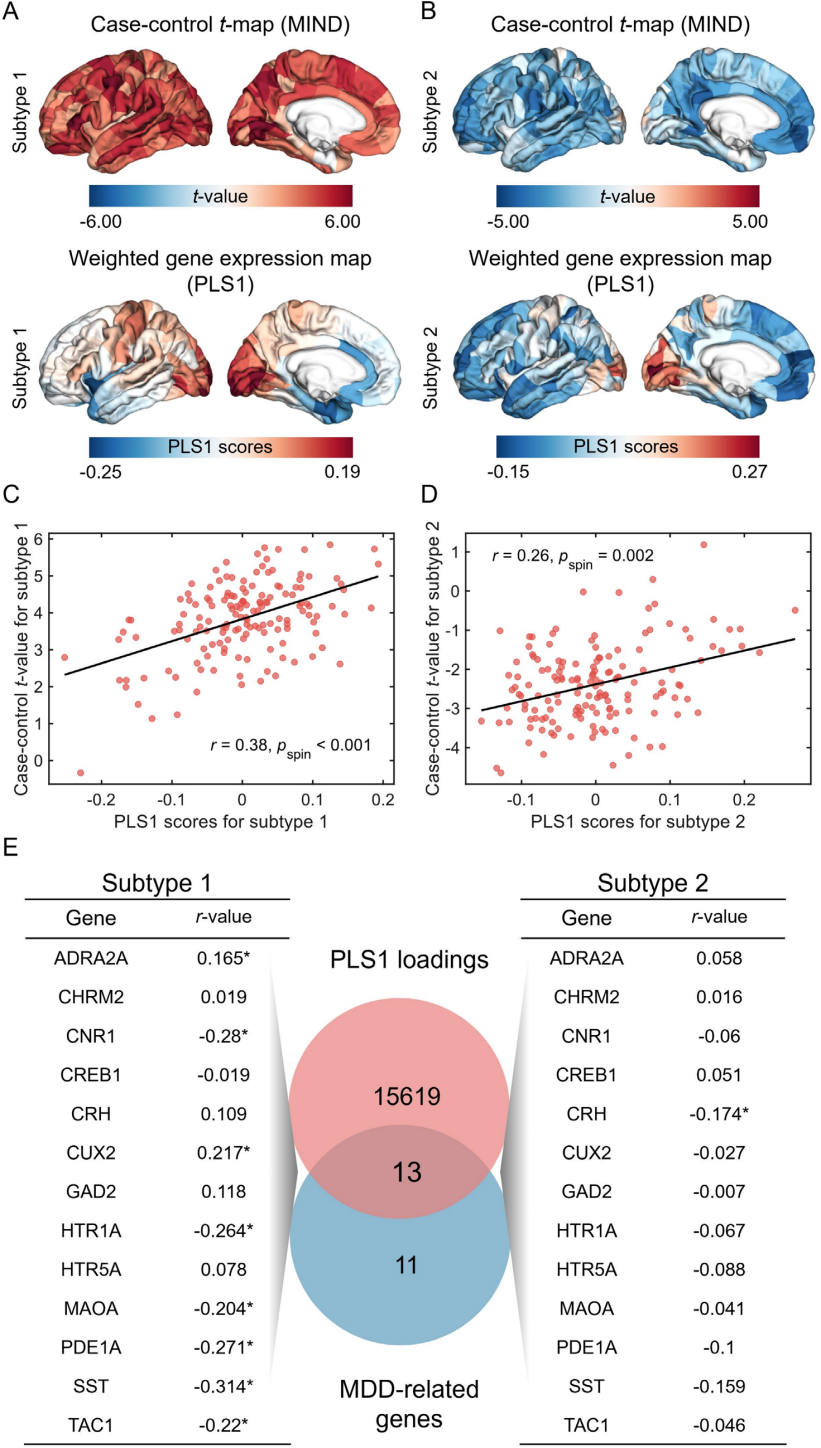
Transcriptomic profiles linked to MIND alterations. (A) Spatial distribution of MIND alterations and PLS1 component mapping within the left hemisphere for MDD subtype 1. (B) Spatial distribution of MIND alterations and PLS1 component mapping within the left hemisphere for MDD subtype 2. (C) Relationship between PLS1 values and MIND alterations in subtype 1 (*r* = 0.38, *p*_spin_ < 0.001). (D) Relationship between PLS1 values and MIND alterations in subtype 2 (*r* = 0.26, *p*_spin_ = 0.002). (E) ISH‐derived MDD‐associated transcript expression demonstrated positive or negative correlations with regional MIND alterations.

PLS regression analyses were conducted with the transcriptional matrix to identify transcriptomic profiles that corresponded to distinct spatial patterns observed in *t*‐maps across MDD subtypes. Thus, the PLS1 successfully explained 23% and 18% of the macrostructural differences between patients with MDD subtypes 1 and 2, respectively (*p*_spin_ < 0.001). PLS1‐derived spatial patterns demonstrated anterior–posterior molecular expression trends in MDD subtypes 1 and 2 (Figure 4A,B). These variations were interpreted as differences in the transcriptional architecture across cortical areas in humans, which are also reflected in the MDD subtype‐related changes observed in the MIND (Burt et al. 2018). Spatial correlation was found between the PLS1‐weighted gene expression profiles and the case–control *t* maps for subtypes 1 and 2 of MDD (subtype 1, *r* = 0.38, *p*_spin_ < 0.001; subtype 2, *r* = 0.26, *p*_spin_ = 0.002; Figure 4C,D). Normalized first component weights were ranked through univariate one‐sample *Z*‐tests. Within MDD subtype 1, analysis revealed 546 positively weighted transcripts and 497 negatively weighted transcripts, with all results showing *p*_FDR_ < 0.05. These positively and negatively weighted gene expressions were associated with increased and reduced regional alterations in MIND. Similarly, in MDD subtype 2, we found 615 PLS1+ genes and 736 PLS1− genes, also with *p*_FDR_ < 0.05, indicating excessive expression and insufficient expression corresponding to regional MIND changes. In total, 1043 genes were identified for MDD subtype 1 and 1351 genes for subtype 2.

To explore the association between gene expressions related to MDD subtypes and regional alterations in MIND, we initially identified 24 genes related to MDD by using the term “depression” of the AHBA ISH data (Hawrylycz et al. 2012; Zeng et al. 2012). From these, we selected 13 genes that overlapped with the 15,632 background genes. In MDD subtype 1 patients, 8 of the 13 MDD‐related genes significantly correlated with regional MIND changes (all *p*_FDR_ < 0.05; Figure 4E). Positive correlations were found for ADRA2A and CUX2, whereas negative correlations occurred with CNR1, HTR1A, MAOA, PDE1A, SST, and TAC1. In contrast, only the CRH gene demonstrated a negative correlation with MIND changes in MDD subtype 2 patients (*p*_FDR_ < 0.05; Figure 4E).

### 3.6 Enrichment Pathways Associated With Regional Alterations in MIND

**FIGURE 5:**
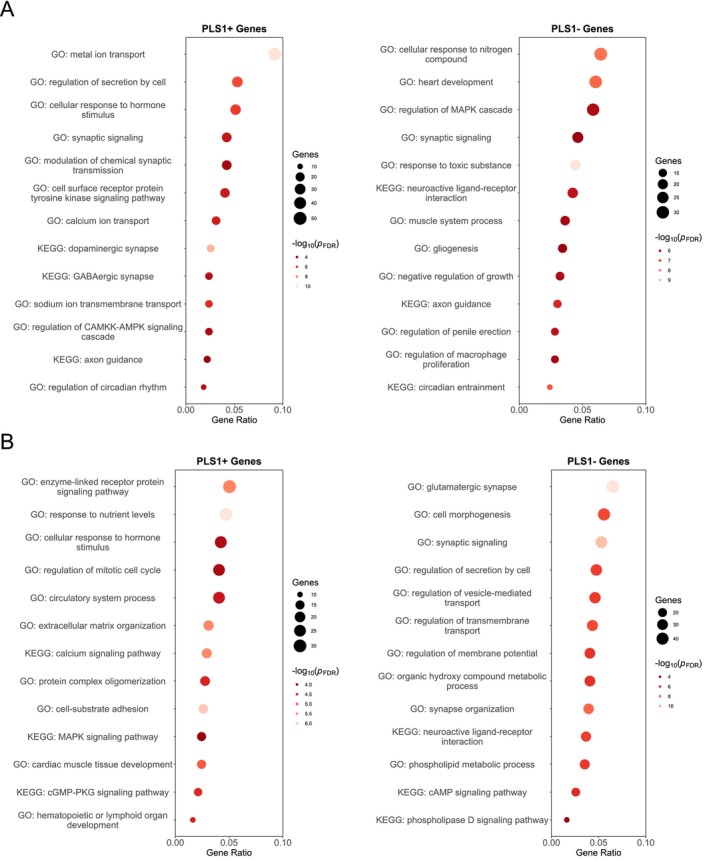
The top 10 enriched pathways from GO and KEGG analyses for PLS1 weighted genes were linked to region‐specific alterations in MIND in subtype 1 (A) and subtype 2 (B).

The GO biological processes and KEGG enrichment analyses of PLS1 weighted genes revealed distinct molecular mechanisms underlying MDD subtypes. In MDD subtype 1, PLS1+ genes demonstrated significant enrichment in metal ion transport, regulation of cellular secretion, hormone‐mediated cellular responses, and synaptic transmission modulation (Figure 5A), while PLS1− genes exhibited primary involvement in nitrogen compound cellular responses, toxic substance responses, and MAPK cascade regulation (Figure 5A). For MDD subtype 2, PLS1+ genes were enriched in enzyme‐linked receptor signaling pathways, nutrient‐level responses, hormone‐mediated cellular processes, and mitotic cell cycle regulation (Figure 5B). The corresponding PLS1− genes showed predominant enrichment in glutamatergic synaptic function, cellular morphogenesis, synaptic signaling networks, secretion regulation, and vesicle‐mediated transport mechanisms (Figure 5B). KEGG pathway analysis revealed subtype‐specific molecular signatures. MDD subtype 1 demonstrated PLS1+ gene enrichment in dopaminergic and GABAergic synaptic pathways alongside axon guidance mechanisms, while PLS1− genes were predominantly associated with circadian entrainment, axon guidance, and neuroactive ligand–receptor interaction networks (Figure 5A). In contrast, MDD subtype 2 exhibited PLS1+ gene involvement in calcium signaling, cGMP‐PKG signaling, and MAPK signaling cascades, whereas PLS1− genes engaged in neuroactive ligand–receptor interactions, phospholipase D signaling, and cAMP signaling pathways (Figure 5B). These findings demonstrate distinct transcriptional architectures underlying MDD heterogeneity, with subtype 1 characterized by metal ion dysregulation and monoaminergic/circadian pathway disruption, while subtype 2 exhibits glutamatergic dysfunction and altered calcium/cAMP signaling cascades.

## 4 Discussion

This study represents the first comprehensive characterization of neuroanatomical subtypes in MDD through the application of the MIND method coupled with elucidation of molecular mechanisms. The semi‐supervised clustering approach successfully identified two distinct MDD subtypes with contrasting neurobiological profiles: (i) the MIND‐based subtypes demonstrated opposing patterns of cortical morphometric alterations, with subtype 1 exhibiting widespread increases, whereas subtype 2 showed localized decreases in MIND strength; (ii) the subtypes were associated with differential neurotransmitter distribution patterns, with subtype 1 linked to broad dysregulation across serotonergic, dopaminergic, and GABAergic systems, while subtype 2 specifically involved glutamatergic, endocannabinoid, and dopaminergic systems; and (iii) the transcriptomic analysis revealed subtype‐specific gene expression signatures, including distinct MDD‐related genes (SST and CUX2 for subtype 1; CRH for subtype 2) and pathway enrichments spanning neurotransmitter system dysregulation, synaptic plasticity mechanisms, cellular homeostasis pathways, and circadian rhythm networks. These findings provide novel molecular‐level insights into MDD neurobiological heterogeneity and establish transcriptional foundations for precision psychiatric approaches targeting subtype‐specific therapeutic interventions.

### 4.1 Neuroanatomical Heterogeneity Captured by MIND Alterations

While recent studies have reported abnormal MIND patterns in patients with MDD, indicating increased changes in MIND across various cortex regions (Gong et al. 2025; Xue et al. 2025), these findings may be influenced by the clinical heterogeneity within MDD cohorts. We identified two distinct subtypes of MDD with abnormally opposite MIND patterns, revealing significant neurophysiologic differences between them. Specifically, subtype 1 patients demonstrated increased MIND in cortical areas, including the prefrontal cortex, lateral temporal and occipital regions, and posterior parts of the parietal lobe. These results were consistent with the findings of previous studies (Xue et al. 2025) that have highlighted altered brain morphometric patterns in MDD, particularly in areas involved in cognitive control, emotional regulation, and sensory processing. Nevertheless, subtype 2 patients demonstrated predominantly decreased MIND strength in overlapping but distinct anatomical territories, characterized by reduced MIND strength in several key regions, including the left medial orbitofrontal gyrus, right inferior temporal gyrus, and right posterior cingulate cortex. Decreased MIND values may indicate that these patients manifested altered cytoarchitectural organization and compromised axonal connectivity within specific brain structures (Sebenius et al. 2025), reflecting developmental or acquired disruptions to neural circuit integrity. This bidirectional pattern suggested that MDD encompassed distinct modes of cortical morphometric organization, potentially reflecting different underlying pathophysiological mechanisms.

The MDD‐related changes in MIND strength exhibited distinct patterns of accumulation across specific functional and cytoarchitectonic systems. As has been demonstrated in prior research (Xue et al. 2025), subtype 1 patients presented with increased MIND strength across all seven Yeo functional networks, including visual, somatomotor, attention, limbic, frontoparietal, and default mode networks. This suggested that within subtype 1 patients, MIND strength was widely increased across networks that may possess more efficient axonal connectivity and inter‐network communication, potentially reflecting a compensatory mechanism to maintain cognitive and emotional homeostasis in the face of depressive symptoms (Cao et al. 2021; Li et al. 2021). In contrast, subtype 2 exhibited reduced MIND strength primarily affecting the somatomotor, dorsal attention, limbic, frontoparietal, and default mode networks. These reductions in MIND strength could reflect a disruption in the brain's ability to efficiently communicate across these essential systems, which are implicated in the regulation of motor functions, attentional processes, emotional stability, and self‐referential thinking (Kaul et al. 2021). The decrease in MIND strength within these regions may indicate a more pronounced pathological effect in subtype 2, whereby these individuals experience impaired connectivity that exacerbates the clinical manifestation of MDD. These reductions may underlie the motor and emotional dysfunctions seen in subtype 2, highlighting the more severe neurobiological disruptions in this group. Within the von Economo cytoarchitectonic networks, both subtypes affected the primary motor, association, and limbic networks, but with opposing directionality.

No relationship was observed between MIND strength and symptomatology in individuals with MDD, implying that regional macrostructural irregularities could occur prior to the clinical presentation in the progression of MDD. Although our neuroanatomical subtypes did not show direct correlations with BDI‐II severity scores, this finding does not preclude their clinical relevance. The absence of correlation between MIND alterations and symptom severity suggests that these neurobiological subtypes may reflect distinct pathophysiological mechanisms rather than simple illness severity gradients, consistent with the Research Domain Criteria (RDoC) (Pacheco et al. 2022) framework emphasizing biological over symptom‐based classification. Prospective studies should evaluate whether these neurobiological subtypes predict: (1) differential responses to mechanism‐specific treatments, (2) correspondence with DSM‐5 specifiers (anxious distress, mixed features, melancholic features), and (3) distinct longitudinal trajectories and functional outcomes. Validation of these hypotheses could establish neuroimaging‐based biomarkers for precision psychiatry, transforming depression treatment from symptom‐based to mechanism‐guided approaches. Our study provided complementary insights to existing MDD subtyping approaches. While Tang et al. (2025) identified the amplitude of low‐frequency fluctuations‐based functional subtypes using K‐means clustering, our HYDRA‐based analysis of MIND networks revealed two distinct structural similarity subtypes with fundamentally different neuroanatomical patterns. Our subtypes exhibited directional patterns, offering more interpretable neuroanatomical phenotypes. Our integration with neurotransmitter receptor density maps directly implicated specific neurotransmitter systems, providing mechanistically informed therapeutic targets. While Tang et al. (2025) emphasized inflammatory and genetic signatures through multi‐omics profiling, our transcriptomic analysis revealed distinct molecular pathways (SST/CUX2 vs. CRH signaling) that may capture orthogonal aspects of MDD heterogeneity. These findings suggest that structural similarity and functional neuroimaging approaches identify complementary dimensions of MDD heterogeneity.

Our analyses have highlighted distinct alterations in brain network coordination and specialization within MDD subtypes. Specifically, subtype 1 exhibited a positive spatial correlation with control MIND patterns, while subtype 2 showed a negative correlation, suggesting disruptions in both coordination and specialization between brain regions. These findings imply decoupling and dedifferentiation, indicative of dysfunction in cortical organization and impaired brain network integrity.

### 4.2 Patterns of Neurotransmitter System Dysregulation Between MDD Subtypes

The associations between MIND alterations and neurotransmitter distributions revealed subtype‐specific neurochemical signatures that provided insights into the underlying mechanisms of morphometric alterations. In MDD subtype 1, extensive associations with multiple neurotransmitter systems suggested a complex, bidirectional neurochemical profile. The negative associations with postsynaptic dopamine receptors (D1 and D2) and dopamine transporter (DAT), alongside positive associations with dopamine synthesis (FDOPA), indicated a compensatory upregulation of dopaminergic signaling in response to reduced dopamine production capacity in MDD (Mizuno et al. 2023). The significant negative association between GABAa receptor availability and MIND activation patterns in MDD subtype 1 aligned with established neurobiological evidence of GABAergic dysfunction in depression. Previous studies have consistently demonstrated reduced GABA concentrations and altered GABAergic interneuron function in limbic and prefrontal regions of MDD patients (Thompson 2024), suggesting that diminished inhibitory neurotransmission contributes to depressive pathophysiology. This negative correlation indicates that regions with greater case–control differences exhibit correspondingly lower GABAa receptor density, providing mechanistic support for GABAergic hypofunction as a core feature of this MDD subtype's neural circuit abnormalities.

Neurotransmitter receptor associations revealed complex subtype‐specific patterns that diverged from established MDD neurochemistry findings. The positive associations with the 5‐HT2A receptor in subtype 1 of MDD contrast with postmortem evidence of reduced 5‐HT2A binding in the auditory cortex of MDD patients (Steinberg et al. 2019). The divergence from prior findings may in part reflect regional specificity, given that our analysis encompassed multiple cortical areas implicated in mood regulation, whereas the earlier study was confined to the auditory cortex. Recent PET evidence using [^11^C]Cimbi‐36 demonstrated reduced 5‐HT release capacity in patients experiencing major depressive episodes (Erritzoe et al. 2023), providing direct evidence for deficient serotonin release in major depressive episodes. This chronic serotonin depletion likely triggered compensatory upregulation of postsynaptic 5‐HT2A receptors through transcriptional activation of HTR2A gene expression via c‐Fos and Egr‐1 pathways (Jaggar et al. 2017). The subtype‐specific 5‐HT2A upregulation, concurrent with dopaminergic hypofunction in subtype 1, suggests severe neurotransmitter deficits requiring stronger compensatory responses.

Meanwhile, the positive associations with the SV2A receptor in MDD subtype 1 contrast with established findings that demonstrated an inverse correlation between the severity of depression and SV2A density (Holmes et al. 2019). This divergent pattern suggests preserved synaptic vesicle machinery in subtype 1, potentially indicating a distinct neurobiological phenotype. Taken together, these findings highlight a multifaceted neurochemical remodeling process in MDD subtype 1, characterized by compensatory adaptations in response to extensive cortical structural alterations. These relationships provide evidence for heterogeneous neurochemical mechanisms underlying MDD subtypes, supporting the existence of molecularly distinct depression variants with preserved compensatory mechanisms.

MDD subtype 2 patients exhibited associations with a distinct neurochemical profile characterized by serotonergic (5‐HT1a), endocannabinoid (CB1), dopaminergic (D1, FDOPA), GABAergic (GABAa), and glutamatergic (NMDA, mGluR5) systems. The prominent involvement of glutamatergic signaling represented a critical pathophysiological distinction, as glutamate‐GABA interactions were fundamental to cortical excitatory‐inhibitory (E/I) balance maintenance in MDD (Hu et al. 2023). The concurrent alterations in GABAergic (GABAa) and glutamatergic (NMDA, mGluR5) signaling suggested a systematic disruption of E/I homeostasis, where impaired glutamate‐mediated excitation and GABA‐mediated inhibition may have contributed to the observed cortical MIND reductions. The association with CB1 endocannabinoid receptors suggested that the CB1 receptor may help mitigate morphological alterations by maintaining neurovascular health and reducing the neuroinflammatory response in depression (Dudek et al. 2025), which was consistent with previous research (Xue et al. 2025). The specific involvement of mGluR5 provided additional mechanistic insight, as this receptor subtype regulated synaptic plasticity, long‐term potentiation, and homeostatic scaling processes essential for cortical stability (Ramos‐Prats et al. 2024). The opposing dopaminergic trends observed between MDD subtype 1 (D1 and D2) and subtype 2 (D1) suggest subtype‐specific dysregulation within cortico‐limbic circuits (H. Chen et al. 2024). In subtype 1, concurrent reductions in D1‐ and D2‐associated binding may underlie the impaired relay of information through the cortico‐striatal‐pallido‐thalamic network involved in this disorder (Hamilton et al. 2018). By contrast, subtype 2 exhibited selective D1 upregulation despite unchanged or opposing D2 activity, likely reflecting postsynaptic sensitization to sustain prefrontal excitatory drive under presynaptic insufficiency, as supported by reduced FDOPA uptake (Grace 2016). Mechanistically, stress‐induced glucocorticoids downregulate D2 receptors via β‐arrestin internalization (Jiang et al. 2022), whereas chronic dopamine depletion upregulates D1 receptors through cAMP–PKA signaling (De Risi et al. 2025). These contrasting receptor dynamics underscore the need for subtype‐tailored dopaminergic interventions. The disruption of these glutamatergic mechanisms may have underlain the systematic morphometric reductions observed in MDD subtype 2 patients.

Treatment‐resistant depression (TRD) represents a complex clinical phenomenon affecting 30%–40% of MDD patients who fail to respond to adequate antidepressant trials (McIntyre et al. 2023). Rather than considering TRD as a distinct diagnostic subtype, our findings suggest it may emerge from the underlying neurobiological heterogeneity we identified through MIND‐based clustering. The two neuroanatomical subtypes demonstrate fundamentally different patterns of brain alterations and molecular mechanisms, which may contribute to differential treatment response profiles. Our identified subtypes exhibited distinct neurobiological characteristics that may predispose individuals to treatment resistance through different pathways. This multisystem dysregulation within subtype 1 may contribute to treatment resistance because standard monoaminergic antidepressants may be insufficient to address the complex, multi‐neurotransmitter dysfunction pattern, while subtype 2 may be predisposed to treatment resistance because conventional monoaminergic antidepressants do not directly address the glutamate‐GABA excitatory‐inhibitory imbalance. This perspective shifts our understanding from viewing TRD as treatment failure to recognizing it as a potential indicator of neurobiological subtype‐treatment incompatibility.

### 4.3 Subtype‐Specific Molecular Mechanisms Revealed by Transcriptional Architecture

Transcriptomic analysis revealed distinct gene expression profiles specific to each MDD subtype. The anterior–posterior gradient of gene expression alterations in both subtype 1 and subtype 2 suggests that these subtypes involve fundamentally different changes to the transcriptional organization of the cortex. In MDD subtype 1, the positive associations between MIND changes and the expression of ADRA2A and CUX2 genes suggest that brain structural alterations might have been driven by specific molecular pathways. ADRA2A mediated presynaptic norepinephrine regulation and stress response modulation (Ali and Dwivedi 2025), while CUX2‐dependent transcriptional programs regulated dendritic complexity and spine density in cortical pyramidal neurons (Singh et al. 2024). These processes directly impacted regional gray matter volume and cortical thickness measurements. In addition, in MDD subtype 1, MIND changes were negatively correlated with the expression of PDE1A and SST genes. PDE1A, which regulates cyclic nucleotides crucial for neuronal signaling and synaptic plasticity (Samidurai et al. 2021), and SST, a neuropeptide expressed in inhibitory interneurons that modulates cortical excitability (Luo et al. 2025), both played key roles in shaping brain network properties, offering insights into the underlying mechanisms of morphometric disruptions in this subtype.

The association of the CRH gene with MIND alterations in subtype 2 underscored the role of hypothalamic–pituitary–adrenal axis dysregulation in this subtype. Negative *t* values from spatial correlation analysis revealed elevated CRH expression in regions with significant structural deviations, highlighting an inverse relationship between corticotropin signaling intensity and neuroanatomical integrity. This pattern suggested chronic HPA hyperactivation as the primary mechanism driving subtype 2 MIND alterations, with elevated CRH leading to glucocorticoid‐mediated neurotoxic effects (Wiest et al. 2025). These molecular processes result in synaptic pruning and structural compromise (Wiest et al. 2025), impacting cortical thickness and subcortical volumes. Thus, subtype 2 represented a unique variant, reinforcing the view of MDD as comprising diverse subtypes with distinct molecular–anatomical profiles.

Our transcriptomic analysis revealed distinct neurobiological architectures underlying MDD heterogeneity, with subtype‐specific enrichment patterns demonstrating fundamentally different pathophysiological mechanisms. MDD subtype 1 exhibited metal ion dysregulation and monoaminergic pathway disruption, with PLS1+ genes predominantly enriched in dopaminergic and GABAergic synaptic transmission pathways. Dysregulated metal ion homeostasis, including copper and iron metabolism, has been increasingly recognized as contributing to depressive symptomatology through oxidative stress and neuroinflammatory processes (Caro‐Ramírez et al. 2025; Ni et al. 2018). The enrichment of dopaminergic pathways aligned with classical monoamine deficiency theories of MDD (Fries et al. 2023), suggesting that subtype 1 patients might have demonstrated enhanced responsiveness to conventional antidepressants targeting monoaminergic systems. The enrichment of circadian pathway dysregulation may contribute to desynchronization between central and peripheral biological clocks, potentially exacerbating metabolic dysfunction and stress hormone irregularities that perpetuate depressive symptomatology (Mehrhof and Nord 2025). Conversely, MDD subtype 2 demonstrated glutamatergic dysfunction as the primary pathophysiological mechanism, with PLS1− genes significantly enriched in glutamatergic synaptic functions and associated signaling cascades. The predominant involvement of calcium and cAMP signaling pathways suggested altered excitatory neurotransmission and downstream second messenger systems (Fries et al. 2023). This glutamatergic dysregulation pattern might have indicated preferential therapeutic responsiveness to glutamate‐modulating agents, including NMDA receptor antagonists. The enrichment of neuroactive ligand–receptor interactions across both subtypes, albeit with different weighting patterns, suggested shared pathological mechanisms involving neurotransmitter system dysregulation (Voss et al. 2023). These findings advanced our understanding of MDD heterogeneity and provided molecular frameworks for developing subtype‐specific therapeutic interventions targeting distinct neurobiological pathways.

## 5 Strengths and Limitations

This study presents a novel approach to understanding MDD by identifying distinct neuroanatomical subtypes based on multiscale MIND alterations. Through detailed spatial analyses, we demonstrated that MDD patients could be effectively categorized into two subtypes, each exhibiting unique patterns of MIND alterations, neurotransmitter associations, and gene expression profiles. These findings offer insights into MDD heterogeneity and demonstrate that integrating MIND with neurotransmitter and gene expression patterns reveals its neurobiological basis. However, the study has several limitations that warrant consideration. Firstly, the cross‐sectional design limits causal inferences, and the absence of longitudinal data restricts our ability to observe the temporal dynamics of MIND alterations in MDD subtypes. Secondly, the AHBA transcriptomic dataset, obtained from postmortem tissue of six individuals, constrained the capacity to investigate molecular‐neuroimaging correlations between cohorts and potentially overlooked subject‐specific variations. Furthermore, transcriptional information was accessible only from the left hemisphere, indicating that our findings based on gene expression profiles may not comprehensively capture whole‐brain alterations. Finally, the absence of correlations between MIND alterations and clinical symptom severity may reflect the limitations of current symptom‐based measures in capturing neurobiological heterogeneity. Future studies should incorporate more comprehensive clinical phenotyping, including cognitive, behavioral, and functional assessments (Zhang et al. 2025), to better characterize the clinical relevance of neurobiological subtypes.

## 6 Conclusions

In summary, MDD exhibits neurobiologically distinct subtypes characterized by opposing cortical morphometric organization patterns. Using HYDRA clustering methodology, we identified two neuroanatomical subtypes based on the MIND. Subtype 1 demonstrated increased MIND strength with multi‐neurotransmitter system alterations and associations with multiple MDD‐related genes (SST and CUX2), while subtype 2 exhibited reduced MIND strength linked to glutamatergic dysfunction and selective MDD‐related gene involvement (CRH). These findings establish neuroimaging‐based stratification potential for precision psychiatry approaches, offering mechanistically informed therapeutic targeting through integrated MIND, transcriptional, and neurotransmitter profiling in MDD.

## Conflicts of Interest

The authors declare no conflicts of interest.

## Supporting information


**Table S1:** Demographic characteristics of participants included in the sites.
**Table S2:** Imaging protocols for structural MRI in the OPEN SRPBS Multi‐disorder MRI Datasets.
**Figure S1:** Mean MIND distributions of 308 brain regions in MDD subtype 1 and 2 patients and HC. MDD subtypes 1 and 2 and HC groups displayed higher values predominantly in the frontal and temporoparietal cortices, while the insular cortex exhibited lower values.
**Figure S2:** Case–control comparison of regional MIND between MDD subtype 1 patients and subtype 2 patients. The MDD subtype 1 patients showed significantly increased regional MIND across the cortex compared to patients with subtype 2.
**Figure S3:** Subtyping results with HYDRA. The results showed that the Adjusted Rand Index peaks in all samples, split1, and split2 when the number of subtypes was 2.
**Figure S4:** MIND strength differences between each MDD subtype and HC for *K* = 2 in Split1 (left column) and Split2 (right column).
**Figure S5:** The number of overlaps assigned to the same MDD subtype using the leave‐one‐site‐out strategy.

## Data Availability

The data underlying the results of this study can be accessed through the OPEN Strategic Research Program for Brain Sciences (SRPBS) Multi‐disorder MRI database, available at https://doi.org/10.1038/s41597‐021‐01004‐8.
