# Supplementary material for: Molecular Mechanisms Explaining Neuroanatomical Subtypes in Major Depressive Disorder: Insights From Cortical Morphometric Inverse Divergence
Source: Hum Brain Mapp. 2025 Oct 31;46(16):e70383. doi: 10.1002/hbm.70383 (PMC12576961; doi:10.1002/hbm.70383)
Supplement: Supplementary file 1 — Table S1: Demographic characteristics of participants included in the sites. Table S2: Imaging protocols for structural MRI in the OPEN SRPBS Multi‐disorder MRI Datasets. Figure S1: Mean MIND distributions of 308 brain regions in MDD subtype 1 and 2 patients and HC. MDD subtypes 1 and 2 and HC groups displayed higher values predominantly in the frontal and temporoparietal cortices, while the insular cortex exhibited lower values. Figure S2: Case–control comparison of regional MIND between MDD subtype 1 patients and subtype 2 patients. The MDD subtype 1 patients showed significantly increased regional MIND across the cortex compared to patients with subtype 2. Figure S3: Subtyping results with HYDRA. The results showed that the Adjusted Rand Index peaks in all samples, split1, and split2 when the number of subtypes was 2. Figure S4: MIND strength differences between each MDD subtype and HC for K = 2 in Split1 (left column) and Split2 (right column). Figure S5: The number of overlaps assigned to the same MDD subtype using the leave‐one‐site‐out strategy. [file HBM-46-e70383-s001.docx]

Molecular mechanisms explaining neuroanatomical subtypes in major depressive disorder: insights from cortical Morphometric Inverse Divergence

Yao Ge^1^, Lijuan Chen^1^, Yan Bai^1^, Wei Wei^1^, Yu Shen^1^, Kaixin Li^2^, Mengzhu Wang^2^, and Meiyun Wang^1,3*^

^1^Department of Radiology, Zhengzhou University People’s Hospital & Henan Provincial People’s Hospital, Zhengzhou 450003, China.

^2^MR Research Collaboration, Siemens Healthineers Ltd., Beijing 100102, China.

^3^Biomedical Research Institute, Henan Academy of Sciences, Zhengzhou 450003, China.

***Corresponding Author:**

Meiyun Wang

E-mail: mywang@zzu.edu.cn

**Ethics approval and consent to participate**

Ethics approval for SRPBS Multi-disorder MRI database: All participants across the datasets provided informed written consent. The recruitment processes and experimental protocols received approval from the institutional review boards of the lead researchers’ institutions.

**Supplementary methods**

**The split-sample validation**

The multiple repeated splits were employed: 100 random 80-20% train-test splits were performed to assess clustering stability. Regarding label permutation, we implemented a Hungarian algorithm-based label alignment procedure to address the random initialization issue. Specifically, after each HYDRA run, cluster labels were optimally matched to a reference clustering solution (first split) using maximum overlap assignment, ensuring consistent label assignment across iterations. This procedure prevents artificial inflation of variability due to arbitrary label switching.

**The leave-one-site-out validation**

When the number of clusters was set to 2, the predicted labels of 2 subtypes from all 6 sites using leave-one-site-out were compared with the original assignments obtained by taking all the sites together. The percentage overlap of patients that were assigned to the same subtype was 86.17% (91% in site COI, 85% in site HUH, 84% in site HRC, 79% in site HKH, 88% in site KUT, and 90% in site UTO). Statistical significance was assessed using binomial probability testing against chance-level concordance (50%), yielding p < 0.001. Additionally, we computed 95% confidence intervals (81.21%-91.12%) demonstrating statistical reliability of this reproducibility estimate.

**Correlations between regional MIND strength and clinical measures**

Only at the KUT site protocol, the medication equivalent doses were estimated as follows: Antipsychotics using chlorpromazine (CPZ) equivalent dose for the patients with MDD; and antidepressants using imipramine (IMP) equivalent dose for the patients with MDD. Since we only obtained information on whether the patients took benzodiazepines, we used benzodiazepine as a binarized variable. For patients with MDD, the effect of medication on MIND strength was analyzed, with age, gender, TIV, and handedness included as confounding covariates. The correlations between medication equivalent and regional MIND strength were not significant after FDR correction.

**Supplementary Tables and Figures**

Table S1. Demographic characteristics of participants included in the sites.

| Hub Institute | Site(scanner) | Site Label | HC  (Age, y) | MDD  (Age, y) |
| --- | --- | --- | --- | --- |
| Hiroshima University | Center of Innovation at Hiroshima univ (VerioDot, Siemens) | COI | 109, 47.50±11.74 | 69, 43.55±8.35 |
|  | Hiroshima univ hospital (Sigma HDxt, GE) | HUH | 66, 29.51±13.69 | 50, 41.56±9.63 |
|  | Hiroshima Rehabilitation Center (Sigma HDxt, GE) | HRC | 21, 42.57±7.53 | 16, 40.50±8.51 |
|  | Hiroshima Kajikawa Hospital (Spectra, Siemens) | HKH | 29, 45.07±10.21 | 27, 45.96±9.69 |
| Kyoto University  University of Tokyo | Kyoto univ (TimTrio, Siemens)  Univ of Tokyo (MR750W, GE) | KUT  UTO | 61, 34.10±10.81  81, 36.60±11.58 | 16, 42.56±14.31  62, 36.23±11.17 |

**Note:** HC, healthy controls; MDD, major depressive disorder.

Table S2. Imaging protocols for structural MRI in the OPEN SRPBS Multi-disorder MRI Datasets.

| Site | COI | HUH | HRC | HKH | KUT | UTO |
| --- | --- | --- | --- | --- | --- | --- |
| MRI scanner | SIEMENS | GE | GE | SIEMENS | SIEMENS | GE |
|  | VerioDot | Sigma HDxt | Sigma HDxt | Spectra | TimTrio | MR750w |
| Fov, mm | 256 | 256 | 256 | 256 | 240 | 240 |
| Matrix | 256*256 | 256*256 | 256*256 | 256*256 | 240 | 256*256 |
| Voxel size, mm^3^ | 1*1*1 | 1*1*1 | 1*1*1 | 1*1*1 | 0.9*0.9*1 | 1*1*1.2 |
| TR, ms | 2300 | 6812 | 6812 | 1900 | 2000 | 7.7 |
| TE, ms | 2.98 | 1896 | 1896 | 2.38 | 3.4 | 3.1 |
| TI, ms | 900 | 450 | 450 | 900 | 990 | 400 |
| Flip angle, deg | 9 | 20 | 20 | 10 | 8 | 11 |

**Note:** Fov: Field of view; TR, repetition time; TE, echo time; TI, inversion time.


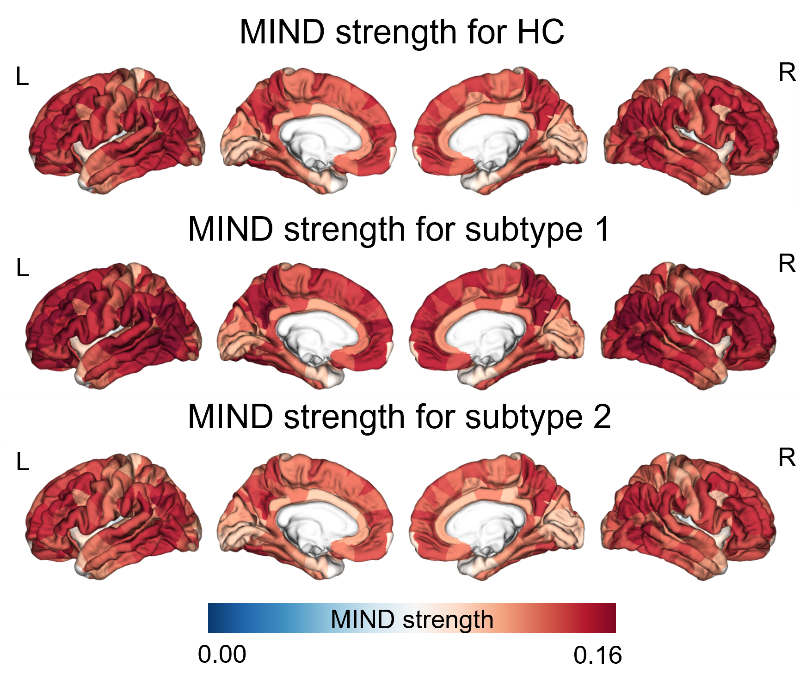


Figure S1. Mean MIND distributions of 308 brain regions in MDD subtype 1 and 2 patients and HC. MDD subtype 1 and 2 and HC groups displayed higher values predominantly in the frontal and temporoparietal cortices, while the insular cortex exhibited lower values.


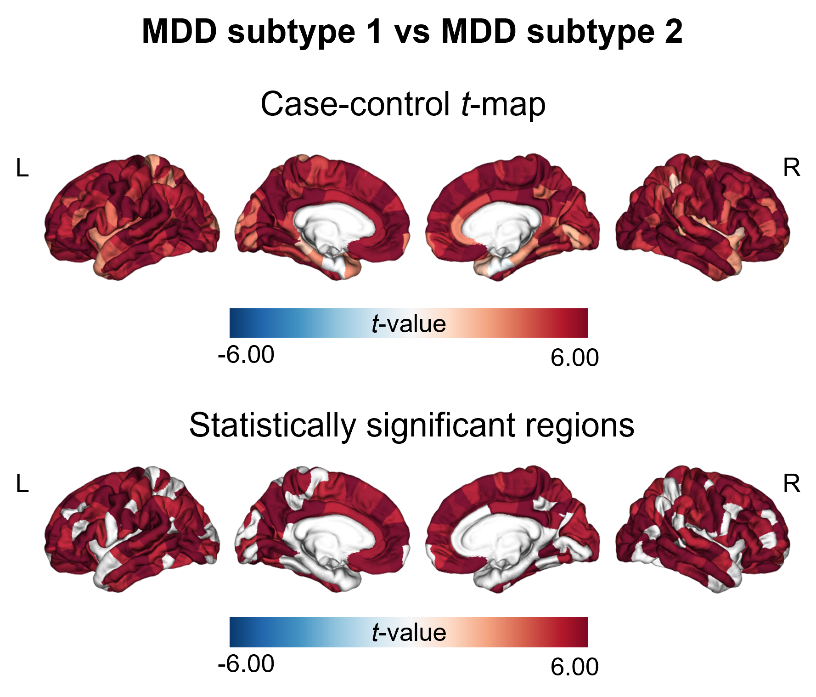


Figure S2. Case-control comparison of regional MIND between MDD subtype 1 patients and subtype 2 patients. The MDD subtype 1 patients showed significantly increased regional MIND across the cortex compared to patients with subtype 2.


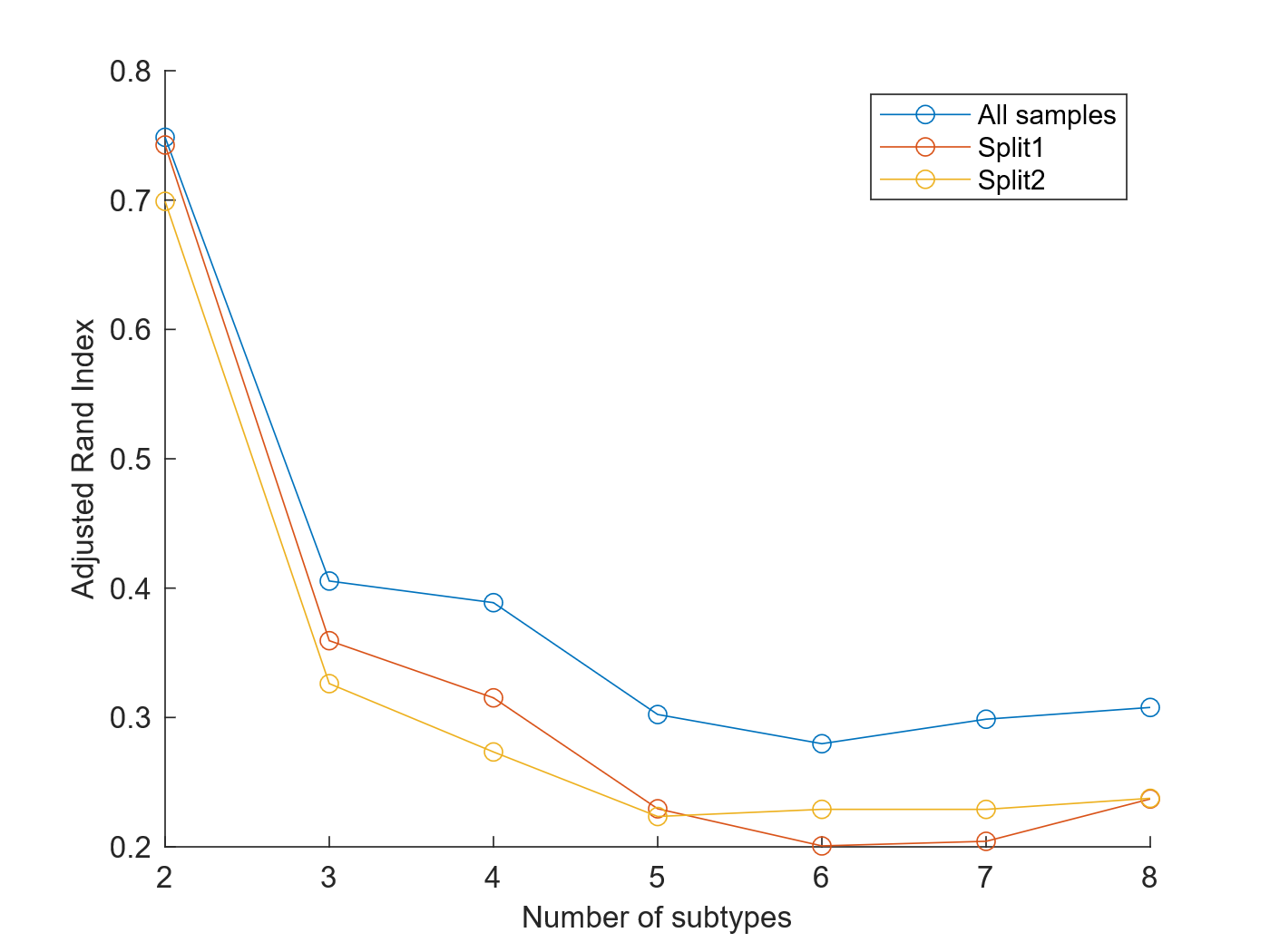


Figure S3. Subtyping results with HYDRA. The results showed that the Adjusted Rand Index peaks in all samples, split1, and split2 when the number of subtypes was 2.


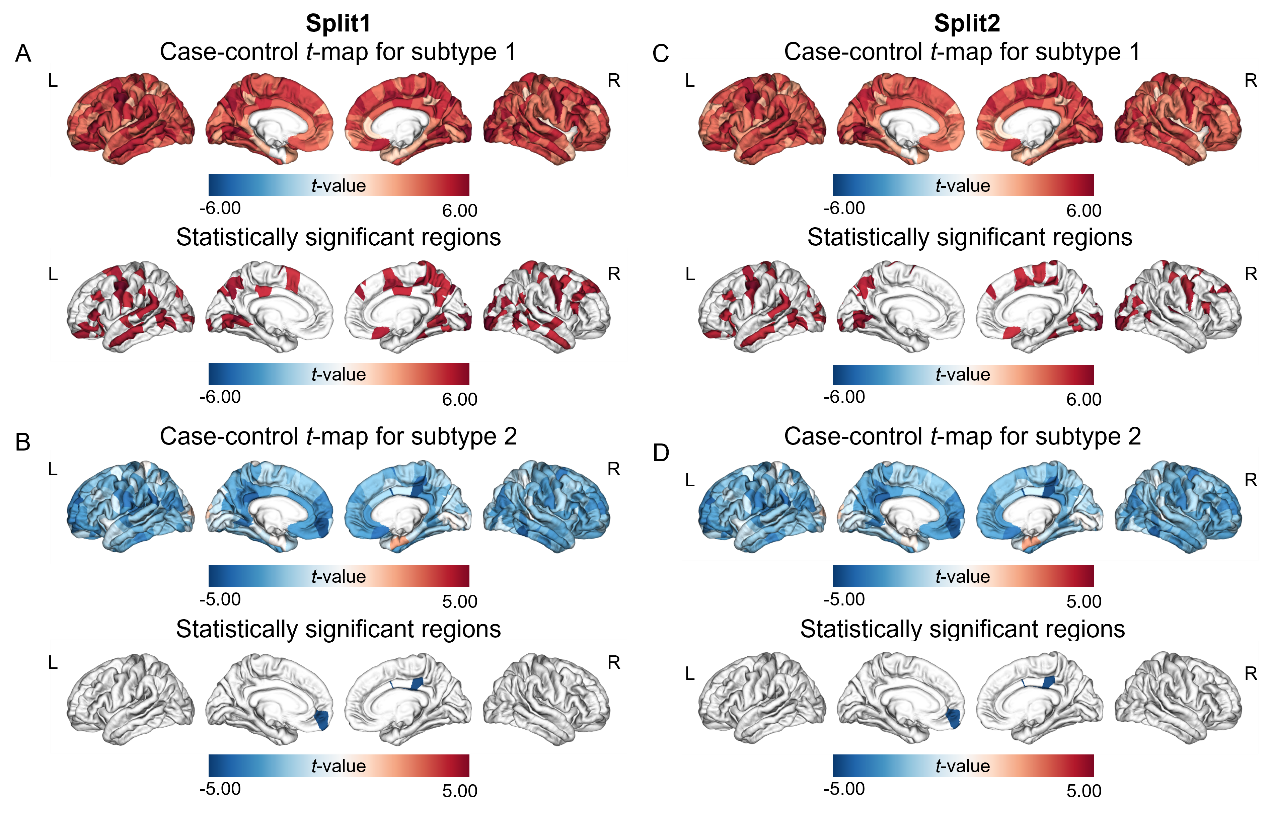


Figure S4. MIND strength differences between each MDD subtype and HC for K = 2 in Split1 (left column) and Split2 (right column).


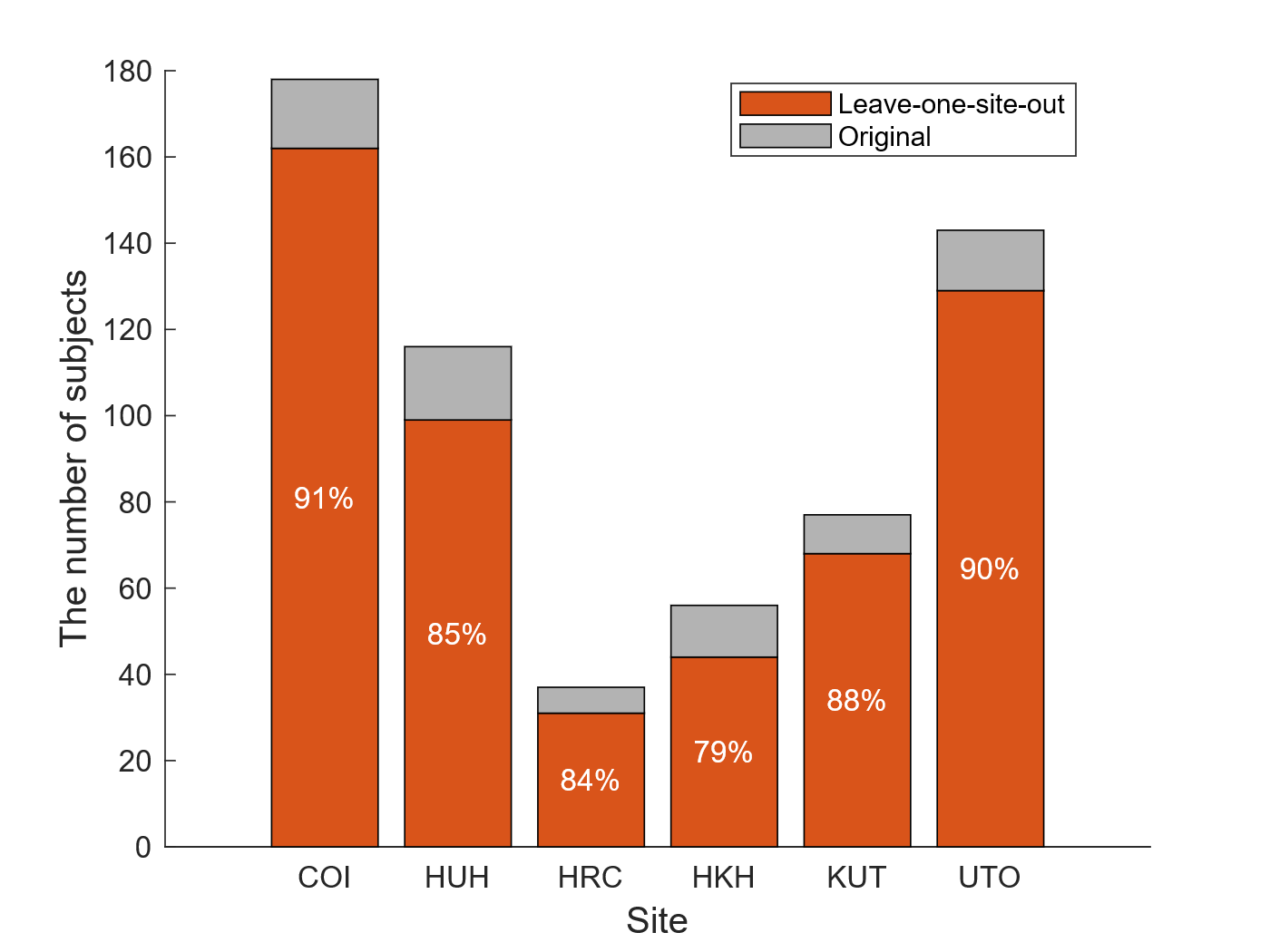


Figure S5. The number of overlaps assigned to the same MDD subtype using the leave-one-site-out strategy.
